# Smoking and Risk of Urolithiasis: Meta-Analysis of Observational Studies

**DOI:** 10.3389/fpubh.2022.816756

**Published:** 2022-03-07

**Authors:** Ling Yue, Qiaofeng Pai, Xiaolin Wu, Jinghua Zhang

**Affiliations:** College of Life Sciences, Henan Agricultural University, Zhengzhou, China

**Keywords:** urolithiasis, smoking, health, meta-analysis, observational

## Abstract

**Background:**

Earlier studies have warned about the effects of smoking on urolithiasis. Some studies have deemed that smoking has a promoting effect on urolithiasis, whereas others have considered that no inevitable association exists between the two. Therefore, we conducted a meta-analysis to estimate whether smoking is associated with urolithiasis risk.

**Methods:**

To identify publications from related observational studies, we performed a search on PubMed, Web of Science, Embase, and the Cochrane Library databases from inception until October 1, 2021. According to the heterogeneity, random-effect model was used to calculate the odds ratios (ORs) and corresponding 95% confidence intervals (CIs).

**Results:**

Five articles were included in the meta-analysis, representing data for 20,402 subjects, of which 1,758 (8.62%) had urolithiasis as defined according to the criteria. Three articles are concerned with analysis between ex-smokers and non-smokers, in which a significant difference was observed (OR = 1.73, 95% CI: 1.48–2.01). Our comparison of current smokers with non-smokers in another meta-analysis of three articles revealed no significant difference between them (OR = 1.08, 95% CI: 0.94–1.23). Finally, we separated subjects into ever-smokers and never-smokers and found a significant difference between the two groups in the analysis of three articles (OR = 1.31, 95% CI: 1.17–1.47). Sensitivity analysis confirmed the stability of the current results.

**Conclusion:**

Combined evidence from observational studies demonstrates a significant relation between smoking and urolithiasis. The trend of elevated urolithiasis risk from smoking was found in ever-smokers vs. never-smokers.

## Introduction

Urolithiasis is one of the most common diseases of the urinary system. It is a global health problem, and its prevalence and incidence are reported to be increasing in the past several decades worldwide, especially in industrialized countries (1–3). It is regarded as a multifactorial disease that involves epidemiological, biochemical, and genetic factors and influences ~12% of the population (4, 5).

Smoking is regarded as one of risk factors of many systemic diseases, such as Alzheimer's disease (6), chronic obstructive pulmonary disease and lung cancer (7), thyroid eye disease (8), and retinal vein occlusion (9). The World Health Organization reported that the number of smokers has increased to almost a billion in 2012 (10) and smoking may lead to roughly seven million deaths by 2020 (11).

The etiology of urolithiasis is complex, and its formation is related to patients' living habits, living environment and individual factors. Epidemiological investigation results show that smoking is related to the occurrence of urolithiasis. The research on its inducing mechanism focuses on the toxicity of cadmium in cigarettes to the kidney.

At present, given that a number of risk factors could result in urolithiasis and the prevalence of urolithiasis and smoking increases rapidly over the years, there seems to be a link between them. Therefore, we conducted a meta-analysis to discuss the relationship between urolithiasis and smoking.

## Materials and Methods

### Literature Search

Until October 1, 2021, full-length and relevant articles were searched from the electronic databases of PubMed, Web of Science, Embase, and Cochrane Library without restriction to languages, regions, or publication types. The related search terms included: “Smoking” [Mesh] and “Urolithiasis” [Mesh]. To find more comprehensive studies, we extended the scope of key words, such as “smoking [Title/Abstract],” “Tobacco [Title/Abstract],” “cigarette [Title/Abstract],” “Urolithiasis [Title/Abstract],” “Nephrolithiasis [Title/Abstract],” “Ureterolithiasis [Title/Abstract],” “Kidney Calculi/Stone [Title/Abstract],” “Renal Calculi/Stone [Title/Abstract],” “Ureteral Calculi/Stone [Title/Abstract],” “Urinary Calculi/Stone [Title/Abstract],” or “Bladder Calculi/Stone [Title/Abstract].” In addition, when multiple articles described the same population, the most complete or newest report was kept.

### Inclusion and Exclusion Criteria

**Figure 1:**
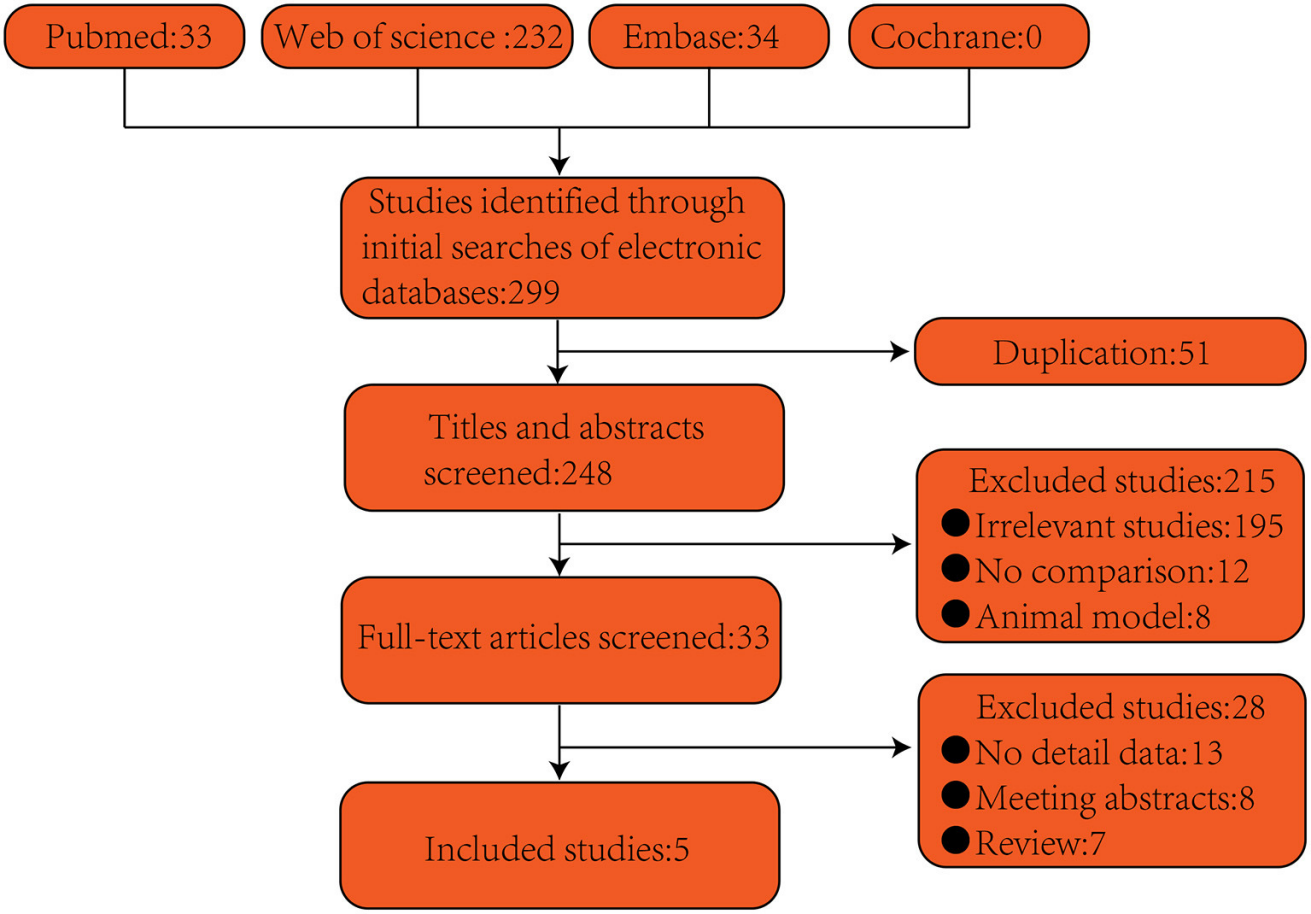
Flow diagram of article selection.

As shown in Figure 1, first, 299 articles (33 articles from PubMed, 232 articles from Web of Science, none from Cochrane, and 34 articles from Embase) were independently searched by two reviewers (LY and QP) according to the above-described methods. Second, duplicates were searched for, 51 articles were excluded consequently. Third, the title and abstract were carefully read, thereby excluding 215 articles. Fourth, the entire texts of the articles were carefully inspected, and 33 articles were excluded consequently. Finally, five studies were eligible and kept according to the criteria: human studies, original research, observational articles, and providing information about associations between urolithiasis and smoking. Odds ratio (OR) estimates are included in the meta-analysis.

### Data Extraction

Two independent investigators (LY and QP) collected the data from all potentially correlative studies. Any conflicting evaluations were solved by the adjudicating senior authors (JZ and XW). Information was extracted from these studies, including name of the first author, publication year, study district, participant age, study design, follow-up time, sample size, amount of urolithiasis with different smoking status, and research quality.

### Heterogeneity Analysis

Sensitivity analysis was performed to better reduce possible heterogeneity among studies.

### Data Synthesis and Analysis

Although smoking histories differ, all participants in most articles were divided into three groups, including ex-smokers, current smokers, and non-smokers. A smoker is someone who is smoking now; non-smokers refer to people who smoke occasionally; ever-smokers refer to people who once smoked and now don't smoke; never-smokers are who have never smoked before and never smokes now.

All statistical analyses were performed using Review Manager 5.3 (Cochrane Collaboration, Oxford, UK) and Stata/SE 12.0 (Stata Corporation, College Station, TX, USA). The odds ratio (OR) were used to compare dichotomous variables, and entire results were reported with 95% confidence interval (CI) (12).

Statistical heterogeneities among articles were assessed using the chi-square test with significance set at *p* < 0.10, and heterogeneity was calculated using the *I*^2^ statistic. Heterogeneity range of 0–40% did not mean importance, 30–60% represent moderate heterogeneity, 50–90% substantial heterogeneity, and 75–100% considerable heterogeneity (13). The random-effects model was used if there was heterogeneity between articles; otherwise, the fixed-effects model was used (14).

### Quality Assessment

Articles were assessed for the level of evidence on the basis of the criteria made by the Center for Evidence-Based Medicine in Oxford, UK (15). The methodological quality of retrospective studies was evaluated by the modified Newcastle-Ottawa scale (16), which includes patient selection, comparability of the study groups, and outcomes assessment. Every article was scored based on the number of stars, and observational studies of over six stars were considered of high quality.

### Ethical Statement

Ethical approval was not necessary as all analyses were based on past studies.

## Results

### Article Selection and Characteristics

Five articles including 20,402 participant (4,066 cases for ex-smokers, 5,457 cases for current smokers, and 10,879 cases for non-smokers) fulfilled the predefined inclusion standard and were considered in the final analysis (Figure 1; Table 1). Five were full-text articles (1, 17–20). Examination of the references listed in these publications did not yield any further research for evaluation. Agreement between the two reviewers was 94% for study selection and 93% for quality.

**Table 1:** Included articles.

**References**	**Publication year**	**District**	**Age scope**	**Study design**	**Definition**	**Case, *N***	**Urolithiasis incidence by smoking status, number/total**			**Research quality**
							**Current smokers**	**Ex-smokers**	**Non-smokers**	
Liu et al. (1)	2009	Taiwan	39–66	CCS	Ultrasonography or radiography	708	123/199	28/58	203/451	⋆⋆⋆⋆ ⋆⋆⋆
Ferraro et al. (17)	2011	American	≥20	RP	Not mentioned	15,690	148/4,114	278/3,876	323/7,700	⋆⋆⋆⋆ ⋆⋆
Hamano et al. (18)	2005	Japan	44–59	CCS	Not mentioned	368	127/174		54/194	⋆⋆⋆⋆
Soueidan et al. (19)	2015	Canada	≥18	RP	Not mentioned	161	12/19	15/51	29/91	⋆⋆⋆⋆
Tang et al. (20)	2015	Guang Xi	≥18	RP	Ultrasonography	3,475	120/951	18/81	280/2,443	⋆⋆⋆⋆ ⋆⋆

*CCS, case-control study; RP, retrospective design*.

### Meta-Analysis Results

#### Ex-smokers vs. Non-smokers

**Figure 2:**
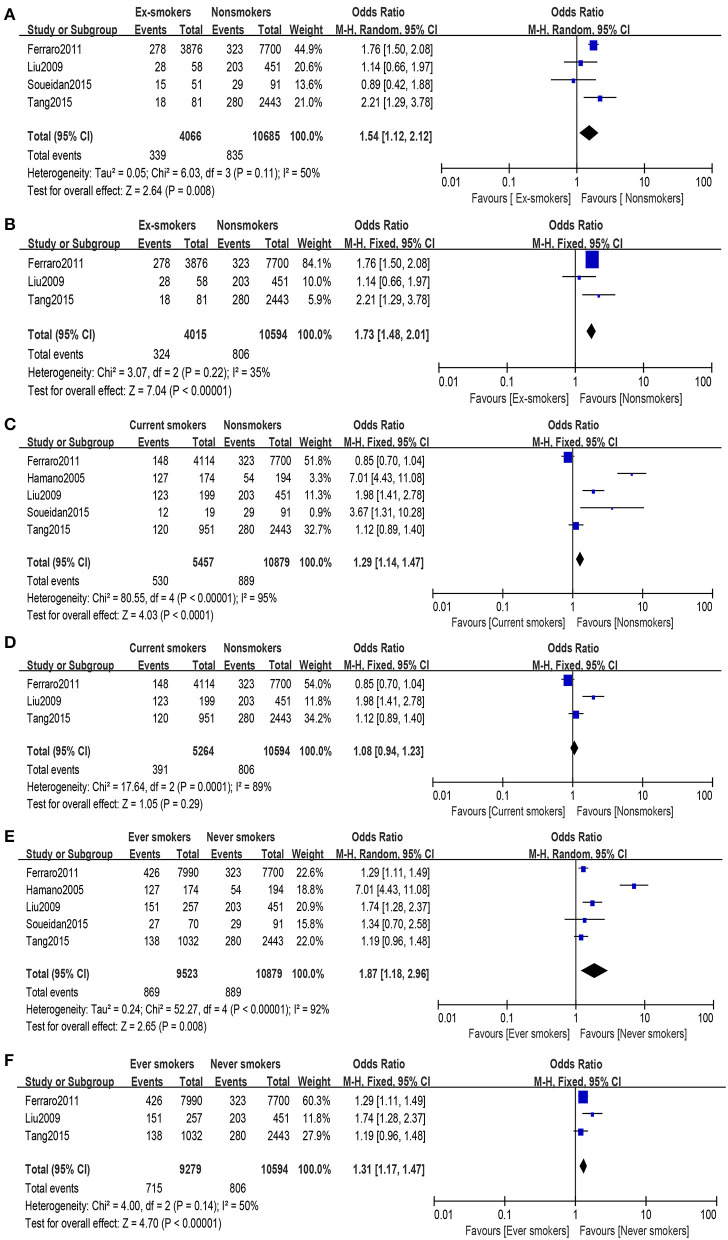
Forest plot: **(A)** comparison of ex-smokers vs. non-smokers and risk of urolithiasis, **(B)** comparison of ex-smokers vs. non-smokers and risk of urolithiasis after eliminating the lowest-quality article, **(C)** comparison of current smokers vs. non-smokers and risk of urolithiasis, **(D)** comparison of current smokers vs. non-smokers and risk of urolithiasis after eliminating the lowest-quality article, **(E)** comparison of ever-smokers vs. never-smokers and risk of urolithiasis, and **(F)** comparison of ever-smokers vs. never-smokers and risk of urolithiasis after eliminating the lowest-quality article.

The heterogeneity (*I*^2^ = 50%, *P* = 0.11) was moderate in this group, so the random-effects model was used to evaluate the OR and its 95% CI. A significant association was found between the two groups (OR = 1.54, 95% CI: 1.12–2.12, Figure 2A). Among these articles, their quality differed. When eliminating the lowest-quality article, heterogeneity (*I*^2^ = 35%, *P* = 0.22) was observed, and a meaningful difference was made between the two groups under random-effects evaluation (OR = 1.73, 95% CI: 1.48–2.01, Figure 2B). No publication bias was found in the analysis of the two groups (ex-smokers and non-smokers: PBegg test = 0.734, PEgger test = 0.334; high-quality articles: PBegg test = 1.000, and PEgger test = 0.700).

#### Current Smokers vs. Non-smokers

A substantial heterogeneity (*I*^2^ = 95%, *P* < 0.00001) was observed; hence, we used the random-effects model and observed that smoking is associated with increased risk of urolithiasis (OR = 1.29, 95% CI: 1.14–1.47, Figure 2C). With regard to the high-quality group (eliminating the lowest-quality article), heterogeneity (*I*^2^ = 89%, *P* = 0.0001) was still high, and no statistically significant relation was found in the group under random-effects evaluation (OR = 1.08, 95% CI: 0.94–1.23, Figure 2D). In the article analysis, no bias was found in the current smokers vs. non-smokers (PBegg test = 0.221, PEgger test = 0.173) and the comparison of the high-quality group (PBegg test = 0.296, PEgger test = 0.138).

#### Ever-Smokers vs. Never-Smokers

Some difference was found between the comparison results of ex-smokers vs. non-smokers and current smokers vs. non-smokers; therefore, all research objects were divided into two groups, namely, ever-smokers and never-smokers. Ex-smokers and current smokers were merged into ever-smokers. In the combined group, heterogeneity (*I*^2^ = 92%, *P* < 0.00001) was observed, and under the random-effects model, a significant difference was found between the two groups (OR = 1.87, 95% CI: 1.18–2.96, Figure 2E). Article quality did not affect the final results under random-effects evaluation results (OR = 1.31, 95% CI: 1.17–1.47, Figure 2F), while heterogeneity (*I*^2^ = 50%, *P* = 0.14) was calculated using random-effects model. After excluding the article by Hamano et al., heterogeneity decreased without affecting the results. When investigating ever-smokers vs. never-smokers and risk of urolithiasis, no publication bias was found (PBegg test = 0.462, PEgger test = 0.446). Furthermore, no publication bias was found in the investigation of high-quality group (PBegg test = 1.000, PEgger test = 0.891).

#### Publication Bias and Heterogeneity Analysis

No significant publication bias was found by group analysis, which was calculated through Begg and Egger tests (Stata 12.0, Stata Corporation). With regard to the heterogeneity analysis, we deleted articles individually to determine the origin of heterogeneity and found that Soueidan et al.'s article was the main branch point in all group comparisons. Furthermore, elimination this article did not change the final result.

## Discussion

In the current meta-analysis, we found that smoking was related with an increased risk of urolithiasis, except for the existing slight insignificant difference when comparing current smokers with never-smokers after removing the lowest-quality articles. To date, controversies still exist regarding the relationship between smoking and urolithiasis. Some researchers consider that smoking has an influence on the formation of urinary calculi (1), whereas others argue that there is no credible evidence to demonstrate that cigarette smoking affects the occurrence urolithiasis (21). However, no meta-analysis was conducted to study the relationship between smoking and urolithiasis. In the present meta-analysis, we combined five original articles published before October 1, 2021, all of which are observational studies, including two controlled clinical trials and three retrospective studies. To compare the effect of smoking on different groups of participants with urolithiasis, participants were grouped as “ex-smokers,” “current smokers,” and “non-smokers.”

Our analysis results of ex-smokers and non-smokers demonstrated that smoking was associated with increased risk of urolithiasis. However, when comparing never-smokers and ever-smokers, the heterogeneity was very high that we could not merge these articles despite applying sensitivity analysis to eliminate the two articles. Thus, we regrouped the participants and divided them into two groups: ever-smokers and never-smokers, and when we compared the two groups, the result also proved that smoking affects the formation of urolithiasis.

In our body, cadmium is mainly derived from food and tobacco smoke (22), and smoke's blood cadmium levels is reported to be ~30–40% higher than that of non-smokers (23). The incidence of urinary calculi in chronic cadmium exposure group was significantly higher than that of the normal population, and there was a dose-response relationship between high blood cadmium and urolithiasis (24). Furthermore, some studies reported that free radicals had a tight association with urolithiasis (25–27).

Urinary calculi are formed through the following stages: nucleus formation, growth, aggregation of crystals, and concretion. The first three stage mainly involve inorganic components and occur in urine and *in vitro* experimental systems, while the last stage occurs in renal tissues, in which organic components are mainly involved (28). The essential part in the formation of urinary stones is crystal adhesion to renal tubular epithelial cells (28). The nucleation process usually occurs in epithelial cells, cell debris, and other crystal surfaces. When broken cell debris is excreted in the urine, the threshold for the concentration of minerals inducing crystal formation decreased; therefore, crystals took shape (26). Damage to renal tubule epithelial cells could significantly promote the crystallization of adhesion (26, 27). Cigarette smoke contains numerous free radicals that greatly damage the kidney (29–32). Thus, smoking can highly lead to the formation of urinary calculi.

Limitations of this meta-analysis should be noted. Individual participant or original data were not available so that our ability was limited to do more detailed analysis. In our search process, we found an article involving a large sample population (21) that did not provide available data for our analysis and was therefore excluded. In addition, insufficient follow-up time could have influenced our conclusions about smoking and urolithiasis. Although these studies are of worldwide origin, data from Europe and African countries are still lacking. In the included articles, no difference between tobacco and cigarette was found; therefore, varied types of smoking were not analyzed as subgroups. Besides, our findings may not be valid enough for generalization in all urolithiasis populations and may not have enough data to evaluate the risk. Moreover, the included articles did not record specific smoking amount and frequency. With limited number of studies conducted, we await more studies about this aspect in the future.

Heterogeneity in some groups is high and noteworthy. First, the observed heterogeneity may be attributed to differences in the chosen standards for smoking. Second, the diagnostic method for urolithiasis varies. The gold standard in examination for urolithiasis is medical imaging; however, not all studies met the criterion. Third, although most of the articles divide the participants into current smokers, ex-smokers, and non-smokers, detailed smoking duration and frequency for each participant are unclear. In the heterogeneity analysis, the article by Hamano et al. may be the principal source. However, we removed this study in our analysis and found no change on the final results. Thus, we kept this article in the meta-analysis.

## Conclusion

Our results indicate a possible significant association between smoking and urolithiasis, and smoking habit may be an independent risk factor in the development of urolithiasis. However, more well-designed studies are still needed to explore the effects of smoking on the risk for urolithiasis.

## Author Contributions

JZ and LY wrote the manuscript. LY and QP collected the data. XW and JZ provided ideas and revised the manuscript. All authors discussed the results and contributed to the final manuscript.

## Funding

This work was supported by grants from the National Natural Science Foundation of China (Grant Number: 32000244) and Key Scientific and Technological Project of Henan (Grant Number: 202102110011).

## Conflict of Interest

The authors declare that the research was conducted in the absence of any commercial or financial relationships that could be construed as a potential conflict of interest.

## Publisher's Note

All claims expressed in this article are solely those of the authors and do not necessarily represent those of their affiliated organizations, or those of the publisher, the editors and the reviewers. Any product that may be evaluated in this article, or claim that may be made by its manufacturer, is not guaranteed or endorsed by the publisher.
